# Experiences and perspectives of healthcare professionals implementing advance care planning for people suffering from life-limiting illness: a systematic review and meta-synthesis of qualitative studies

**DOI:** 10.1186/s12904-023-01176-7

**Published:** 2023-05-06

**Authors:** Nanxi Zhu, Liu Yang, Xianlin Wang, Jinmei Tuo, Liuliu Chen, Renli Deng, Rick Yiu Cho Kwan

**Affiliations:** 1 Nursing Department, Affiliated Hospital of Zunyi Medical University, 121 Dalian Road, Zunyi City, Huichuan District, Guizhou Province 563000 China; 2 Nursing Department, Fifth Affiliated Hospital of Zunyi Medical University, Zhuhai, 519100 China; 3 School of Health, Zhuhai College of Science and Technology, Zhuhai, 519041 China; 4 Tung Wah College, Hong Kong, HKG China

**Keywords:** Advance care planning, Advance directives, Healthcare professional, Palliative care, Systematic review, meta-synthesis

## Abstract

**Background:**

Life-limited patients may lose decision-making abilities during disease progression. Advance care planning can be used as a discussion method for healthcare professionals to understand patients’ future care preferences. However, due to many difficulties, the participation rate of healthcare professionals in advance care planning is not high.

**Aim:**

To explore the facilitators of and barriers to healthcare professionals’ provision of advance care planning to life-limited patients to better implement it for this population.

**Methods:**

We followed ENTREQ and PRISMA to guide this study. We conducted a systematic search of PubMed, Web of Science, Embase, CINAHL, PsycINFO, CNKI, and SinoMed to include qualitative data on the experiences and perspectives of healthcare professionals in different professional fields in providing advance care planning for life-limited patients. The Joanna Briggs Institute Critical Appraisal Checklist for Qualitative Research was used to assess the quality of the included studies.

**Results:**

A total of 11 studies were included. Two themes were identified: unsupported conditions and facilitative actions. Healthcare professionals regarded cultural concepts, limited time, and fragmented record services as obstacles to implementation. They had low confidence and were overly concerned about negative effects. They needed to possess multiple abilities, learn to flexibly initiate topics, and facilitate effective communication based on multidisciplinary collaboration.

**Conclusion:**

Healthcare professionals need an accepting cultural environment to implement advance care planning, a sound legal system, financial support, and a coordinated and shared system to support them. Healthcare systems need to develop educational training programs to increase the knowledge and skills of healthcare professionals and to promote multidisciplinary collaboration to facilitate effective communication. Future research should compare the differences in the needs of healthcare professionals in different cultures when implementing advance care planning to develop systematic implementation guidelines in different cultures.

**Supplementary Information:**

The online version contains supplementary material available at 10.1186/s12904-023-01176-7.

**What is already known about the topic?**

- Patients with life-limiting illnesses are at risk of losing the ability to make end-of-life decisions due to their cognitive or health condition.
- Advance care planning can be used as a discussion method for healthcare professionals to understand patients’ future care preferences.
- Due to many difficulties, the participation rate of healthcare professionals in advance care planning is not high.

**What this paper adds?**

- Healthcare professionals regarded cultural concepts, limited time, and fragmented record services as obstacles to the implementation of advance care planning.
- Healthcare professionals had low confidence and were overly concerned about negative effects.
- Healthcare professionals need to possess multiple abilities, learn to flexibly initiate topics, and facilitate effective communication based on multidisciplinary collaboration.

**Implications for practice, theory, or policy?**

- An acceptable culture, sound legal system, financial support, and a coordinated and shared system are needed to support healthcare professionals in implementing advance care planning.
- Healthcare systems need to develop educational training programs to increase the knowledge and skills of healthcare professionals and promote multidisciplinary collaboration to facilitate effective communication.
- Cultural differences in the needs of healthcare professionals to implement advance care planning should be compared to develop culturally specific and systematic implementation guidelines.

## Introduction

Life-limiting illness (LLI) refers to diseases that have no reasonable hope of a cure [1]. LLI may shorten a person’s life and may include diagnoses of cancer, heart failure, chronic obstructive pulmonary disease, dementia, frailty, chronic liver disease, and kidney disease [2]. At some point in their lives, life-limited patients may experience a high symptom burden, functional decline, and organ failure [3]. Complex decisions about medical care and treatment are often required in life-limiting disease trajectories [4]. Patients are at high risk of losing their ability to make decisions due to their declining health or cognitive function. Thus, patients may not always be treated according to their preferences if healthcare professionals are not clearly informed about their life goals and care preferences [4–6].

Advance care planning (ACP) is a process for individuals, family members, and healthcare professionals that defines and discusses future care goals and preferences, and records and reviews these goals and preferences if appropriate [6]. The value of ACP includes helping people understand their health status and future care options, communicating with their significant others, and identifying their care goals [6]. ACP is viewed as an important strategy to improve end-of-life communication between patients and healthcare professionals and to achieve consistency between preferred and delivered care [7, 8]. Due to the unpredictable but substantial risks of deterioration and death, ACP may be of particular value for patients with progressive diseases [6, 9].

Despite numerous evidence on the positive effects of ACP, the frequency of ACP conversations between patients and healthcare professionals in clinical practice remains low [10–12]. Studies have pointed out that discussing ACP with life-limited patients requires the initiative and effort of healthcare professionals [1]. The reality is that even skilled staff who specialize in palliative care are reluctant to raise the topic and find it difficult to judge when and how to do so [13, 14].

To our knowledge, there is only one review that has summarized the perspectives of patients with LLI on ACP, but it was limited to patients’ perspectives only [1]. Understanding the perceptions and needs of patients is important, as it can help healthcare professionals to provide the services they want. However, ACP is generally controlled by healthcare professionals [15]. Boddy et al. reported that if healthcare professionals are uncertain about ACP, who is responsible for it, and what and how to talk about it, they may not raise these topics with patients [16]. Moreover, if healthcare professionals make sufficient preparations, they can better play the role of introducing ACP, so the opinions of healthcare professionals are equally needed, important, and useful [17]. Their perspectives can reveal existing objective problems about specialization, such as defects in the healthcare system and their urgent need for relevant skills, and thus by examining these problems, the necessary conditions for the long-term development of ACP will also be revealed.

To increase the participation of healthcare professionals in providing ACP for life-limited patients, and to create a coordinated environment for ACP that can benefit everyone, the field needs to identify relevant obstacles and develop effective ways to help clinical practice. Therefore, we systematically integrated healthcare professionals’ experiences and views on providing ACP for life-limited patients to deeply understand their obstacles and needs in implementing ACP, which may point to a direction in how better to practice and give full play to the value of ACP.

## Methods

The Enhancing transparency in reporting the synthesis of qualitative research (ENTREQ) [18] and Preferred Reporting Items for Systematic Reviews and Meta-Analysis (PRISMA) [19] guided the preparation of this article. The Joanna Briggs Institute (JBI) Critical Appraisal Checklist for Qualitative Research was used to assess the quality of the included studies [20]. The protocol for this systematic review was prospectively registered on PROSPERO (CRD42022326238) and can be accessed in full at www.crd.york.ac.uk/prospero/display_record.php?ID=CRD42022326238.

### Search strategy

Seven databases were searched systematically, including PubMed, Web of Science, Embase, CINAHL, PsycINFO, CNKI, and SinoMed. Subject words were searched through PubMed, different expressions of keywords were found by an electronic dictionary, and the retrieval method of combining subject words and free words was adopted. Additional studies were supplemented by tracking the references of the included studies. Keywords identified for retrieving databases included “advance care plan” **AND** “healthcare professional.” The retrieval period was from the construction of the databases to May 2022. The complete search strategy using Embase as an example is shown in Supplement Appendix 1.

### Eligibility criteria

***Inclusion criteria.*** (1) Participants: healthcare professionals, including community medical workers and hospital medical personnel. (2) Phenomena of interest: focused on the views and experiences of providing ACP for life-limited patients. (3) Context: any hospitals, clinics, communities, or palliative care units. (4) Study design: qualitative studies and mixed-method studies with qualitative data describing healthcare professionals’ perceptions of providing ACP to life-limited patients.

***Exclusion criteria.*** (1) Patients who were children or minors, as healthcare professionals mostly spoke with parents; (2) the full text of the study was not found; and (3) studies not published in English or Chinese.

### Data screening and extraction

**Fig. 1:**
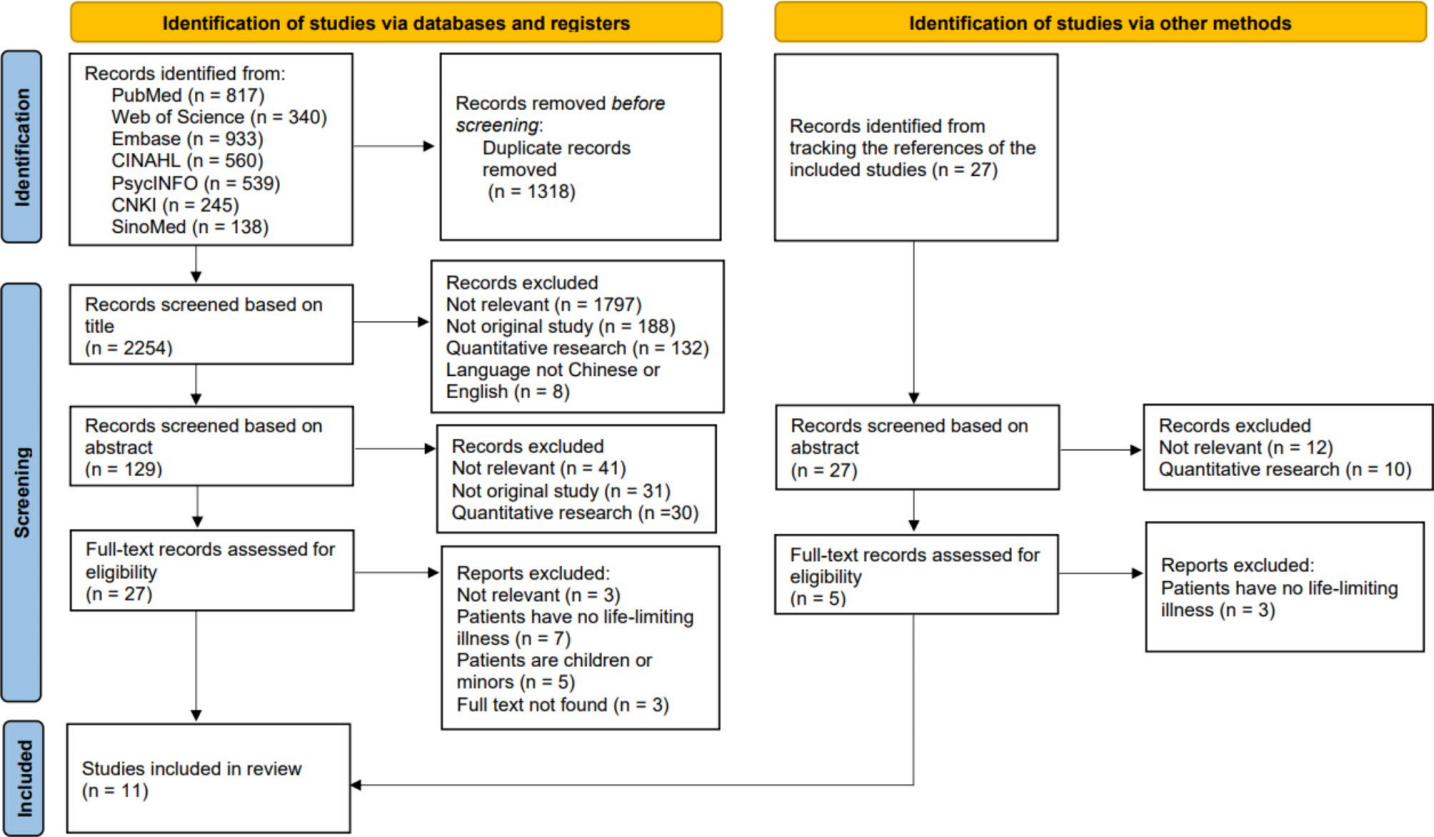
PRISMA flow chart of the study screening process

Screening and study selection were conducted in accordance with PRISMA guidelines, as shown in Fig. 1. EndNote X9 was used to manage all references. After duplication, two authors (NX-Z and LY) screened the titles, abstracts, and full texts against the eligibility criteria independently. After discussion, there were no discrepancies between the two authors. Data extraction was based on the JBI standardized form, combined with the research theme and synthesis method, and decided after discussion. The extracted content was entered into two tables, as shown in Table 1 and Supplementary Appendix 3. Table 1 shows a summary of the studies chosen, including the study (year), country, design, participants, patients’ disease types, care settings, aims, and results. The other table in Supplementary Appendix 3 shows the process of thematic synthesis, including quotations extracted from the included studies. Relevant data were extracted independently by three authors (XL-W, LL-C, and JM-T). For the mixed-method studies, only qualitative data were included. After discussion, there were no discrepancies between the three authors.

**Table 1:** Summary of included studies

Study (Year)	Country	Design	Participants	Patients’ Disease Types	Care Settings	Aims	Themes/Results
Wichmann et al. [27] (2018)	Netherlands	Content analysis; semi-structured interviews; qualitative study	17 GPs	Patients with cancer, heart failure, and COPD	Different clinics across the Netherlands	To identify the experiences of GPs in ACP conversations with palliative patients and the factors affecting these experiences.	Four themes: (1) ACP and society; (2) the role of GPs in ACP; (3) initiating ACP; (4) tailor-made ACP.
Toguri et al. [28] (2020)	Canada	Thematic analysis; descriptive qualitative study using in-depth, semi-structured, one-time, one-on-one interviews	4 patients; 4 family members; 10 oncologists	Advanced cancer patients	Hospital: department of medical oncology and radiation oncology	To explore the understanding, experiences, reflections, and information needs of patients and their families regarding ACP, as well as physicians’ experiences in initiating ACP and their views on ACP training.	Five themes: (1) initiating ACP; (2) the relationship between patient and family influences and the progression of ACP; (3) limited formal training in ACP; (4) ACP requires teamwork; (5) lack of a coordinated health system.
Sellars et al. [29] (2017)	Australia	Grounded theory; thematic analysis; semi-structured interviews; qualitative study	20 nephrologists; 7 nurses; 4 social workers	Patients with chronic kidney disease	Clinics in different regions of Australia with experience in ACP for patients with CKD	To describe the experiences and perspectives of multidisciplinary clinicians regarding ACP, and to provide guidance and recommendations for the implementation of ACP for patients with chronic kidney disease.	Five themes: (1) promoting informed decision-making; (2) ethical challenges across moral boundaries; (3) navigating vulnerable conversations; (4) professional helplessness in initiating ACP; (5) clarified responsibilities.
Schichtel et al. [22] (2021)	United Kingdom	Reflexive thematic analysis; semi-structured interviews; interpretive and descriptive study	17 GPs; 7 nurses	Patients with heart failure	Rural and urban communities: primary health care institutions	To explore the promoting and hindering factors of implementing ACP for patients with heart failure to improve patients’ participation in ACP.	Three main themes: (1) ACP is an integral part of holistic health care in HF; (2) factors that may limit the doctor-patient relationship; (3) approaches to improving patients’ ACP participation.
Robinson et al. [24] (2013)	United Kingdom	Thematic analysis; focus groups and individual interviews; qualitative study	5 specialist palliative care professionals; 10 GPs; 17 community nurses and AHPs; 10 old-age psychiatrists; 22 mental health nurses and AHPs; 6 social workers; 15 ambulance service workers; 3 solicitors; 7 voluntary sector	Dementia patients	Clinical area of palliative care and dementia	To explore professionals’ experiences in the implementation of ACP for dementia and palliative care patients.	Four main themes: (1) the value of ACP; (2) delivering patient choice and achieving desired outcomes; (3) definition and legal issues of ACP; (4) three uncertain aspects of ACP practice: (a) who’s responsibility; (b) when to start; (c) what documentation is used.
O’Hare et al. [25] (2016)	United States	Grounded theory; semi-structured, one-on-one interviews by phone or in person	13 physicians; 6 nurses; 3 dialysis technicians; 2 dieticians; 2 social workers	Patients with advanced kidney disease	Multidisciplinary specialties (geriatric medicine, cardiology, intensive care, nephrology, palliative care, nursing, nutrition, physiatry, primary care, social work, and vascular surgery) at Health Care System	To explore multidisciplinary health professionals’ experiences in and perspectives on ACP for patients with advanced renal disease to determine ways to improve the participation rate of this population.	Four themes: (1) ACP is influenced by clinical setting, the role of different healthcare providers, and disease trajectory; (2) lack of a shared understanding and vision of the relationship between ACP and other relevant forgone life resuscitation discussions; (3) unclear responsibilities; (4) lack of active collaboration and communication.
De Vleminck et al. [30] (2014)	Belgium	Constant comparative analysis; qualitative methodology of focus groups	36 GPs	Patients with cancer, heart failure, and dementia	Palliative home care teams	From GPs’ perspectives, identify the barriers to initiating ACP and gain insight into the barrier differences between ACP trajectories in patients with cancer, heart failure, and dementia.	Two themes: (1) barriers to initiating ACP; (2) differences in ACP initiation barriers in patients with cancer, heart failure, and dementia.
Menon et al. [26] (2018)	Singapore	Explorative qualitative study; focus groups; individual, semi-structured, in-depth interviews; inductive thematic analysis	15 doctors; 13 nurses; 5 medical social workers; 15 patients; 13 caregivers	Patients with LLI	Multiple health care settings: areas such as geriatrics and family medicine tend to care for patients with life-limiting illnesses	To study the attitudes of patients with LLI, informal caregivers, and healthcare professionals (that care for life-limited patients) toward ACP in a multicultural, family-centered community.	Seven themes: (1) ACP may cause loss of hope and/or depression; (2) ACP may not reflect patients’ preferences; (3) family members play an important role in decision-making, especially for elderly patients and those lacking capacity; (4) ACP may burden families; (5) ACP can bring benefits to all stakeholders; (6) society is not ready for ACP; (7) misunderstandings about laws governing medical care decisions for patients with and without capacity.
Manthorpe et al. [31] (2019)	United Kingdom	Semi-structured interviews; framework analysis; exploratory study	7 CPNs; 4 doctors; 3 social workers or occupational therapists	Dementia patients	Community mental health services	To explore community-based healthcare professionals’ understanding of the process, experiences, barriers, and contributing factors of ACP for patients with dementia.	Five themes: (1) ACP knowledge and experience; (2) use of ACP; (3) inhibitors of discussion; (4) service influences; (5) recommendations for future ACP implementation.
Kuusisto et al. [17] (2021)	Finland	Qualitative descriptive study; focus group interviews; individual or couple interviews; inductive content analysis	18 registered nurses; 5 practical nurses; 5 physicians; 5 social workers	Patients with cancer, COPD, and ALS	Palliative care unit in hospital ward or outpatient clinic	To describe medical professionals’ perceptions of ACP in palliative care units in hospital wards or outpatient settings.	Three themes: (1) ACP information content; (2) coordination of ACP care activities; (3) support patients and their families in coping.
Hirakawa et al. [23] (2021)	Japan	Multicenter qualitative study; in-depth semi-structured interviews; content analysis	7 physicians; 23 nurses; 4 care managers; 4 social workers	Patients with severe COPD	Palliative care services	To explore healthcare providers’ perceptions of ACP implementation in adult patients with severe COPD and challenges in facilitating ACP.	Five main themes based on the stakeholder education model: (1) daily decision-making; (2) sense of ethical decision-making; (3) in-depth interviewing skills; (4) collaborative information sharing among team members; (5) dissemination of knowledge about ACP.

ACP = advance care planning; GPs = general practitioners; CKD = chronic kidney disease; HF = heart failure; AHPs = allied health professionals; LLI = life-limiting illness; CPN = community psychiatric nurse; COPD = chronic obstructive pulmonary disease; ALS = amyotrophic lateral sclerosis

### Critical appraisal

This study used the JBI Critical Appraisal Checklist for Qualitative Research to assess the methodological quality of the included studies [20]. The checklist consisted of 10 items, which assessed the research quality in different domains, including research methodology and conceptual depth of reporting. The included studies were rated A if they met the 10-item criteria, which indicated that the quality standards were fully met, and the possibility of bias was remote. They were rated B for meeting one to nine items, which indicated that the quality standards were partially met, and the possibility of bias was moderate. They were rated C for meeting zero items, which indicated that the quality standards were not met at all, so the possibility of bias was high. The evaluation was conducted independently by two authors (NX-Z and LY). Disagreements were resolved through the third author (RL-D) for consultation and judgment.

### Data synthesis

The thematic synthesis technique proposed by Thomas and Harden was used to synthesize the data [21]. This method ensured a clear and transparent link between the conclusion and the text of the preliminary studies. The procedures involved five steps: (1) importing the full text of 11 articles into NVivo 11 Plus; (2) reading and rereading the included studies by three authors (NX-Z, LY, and RL-D) to obtain a preliminary understanding; (3) inductively coding all results and findings line by line according to their meanings by two authors (NX-Z and LY) independently, who then compared their codes during the coding process, and the team met regularly to make iterative improvements to the coding to achieve consensus; (4) looking for similarities and differences among these codes and grouping them into descriptive themes by three authors (NX-Z, LY, and RL-D); and (5) generating the descriptive themes into a set of synthesized findings that resulted in analytical themes by three authors (NX-Z, LY, and RL-D). The synthesized findings were examined by all coauthors.

## Results

### Study selection

As shown in Figs. 1, 3 and 599 articles were obtained after searching the databases and tracking the references of the included studies. After excluding duplicates and screening the titles and abstracts, we reduced the number of papers to 32 for full-text evaluation. Finally, 11 studies fulfilled the eligibility criteria and were included in the meta-synthesis.

### Quality assessment

The quality assessment results of the 11 included studies are shown in Table 2. Only one study was rated A in the quality assessment, while the rest were rated B. Only two studies reported the potential beliefs and values of the researchers that might have influenced the findings [22, 23]. Five studies reported the researchers’ roles in the study that might have potentially influenced the interpretation of the findings [17, 22, 24–26]. The average consistency rate of the two authors after independent evaluation was 0.90, indicating good consistency. The results of each quality assessment item of the 11 articles by the two authors are shown in Supplement Appendix 2.

**Table 2:** Quality assessment of qualitative studies based on the Joanna Briggs Institute Critical Appraisal Checklist

Study	C1	C2	C3	C4	C5	C6	C7	C8	C9	C10	Grade
1. Wichmann et al. [27]	Y	Y	Y	Y	Y	U	U	Y	Y	Y	B
2. Toguri et al. [28]	Y	Y	Y	Y	Y	U	U	Y	Y	Y	B
3. Sellars et al. [29]	Y	Y	Y	Y	Y	U	U	Y	Y	Y	B
4. Schichtel et al. [22]	Y	Y	Y	Y	Y	Y	Y	Y	Y	Y	A
5. Robinson et al. [24]	Y	Y	Y	Y	Y	U	Y	Y	Y	Y	B
6. O’Hare et al. [25]	Y	Y	Y	Y	Y	U	Y	Y	Y	Y	B
7. De Vleminck et al. [30]	Y	Y	Y	Y	Y	U	U	Y	Y	Y	B
8. Menon et al. [26]	Y	Y	Y	Y	Y	U	Y	Y	Y	Y	B
9. Manthorpe et al. [31]	Y	Y	Y	Y	Y	U	U	Y	Y	Y	B
10. Kuusisto et al. [17]	Y	Y	Y	Y	Y	U	Y	Y	Y	Y	B
11. Hirakawa et al. [23]	Y	Y	Y	Y	Y	Y	U	Y	Y	Y	B

C1 = Congruity between the stated philosophical perspective and the research methodology

C2 = Congruity between the research methodology and the research question or objectives

C3 = Congruity between the research methodology and the methods used to collect data

C4 = Congruity between the research methodology and the representation and analysis of data

C5 = There is congruence between the research methodology and the interpretation of results

C6 = Locating the researcher culturally or theoretically

C7 = Influence of the researcher on the research

C8 = Representation of participants and their voices

C9 = Ethical approval by an appropriate body

C10 = Relationship of conclusions to analysis or interpretation of the data

U = unclear; Y = yes

### Study characteristics

The 11 studies were undertaken in the United Kingdom (n = 3), the Netherlands (n = 1), Finland (n = 1), Canada (n = 1), Australia (n = 1), the United States (n = 1), Belgium (n = 1), Singapore (n = 1), and Japan (n = 1). The healthcare professionals included in the studies were general practitioners, oncologists, nephrologists, specialist palliative care professionals, community nurses, allied health professionals, old-age psychiatrists, physicians, dialysis technicians, dieticians, registered nurses, and practical nurses. The care settings in these studies were varied, including urban and rural communities, hospitals, and palliative care units. A summary of the study characteristics is shown in Table 1.

### Findings

Two themes and seven subthemes were identified: unsupported conditions (unsupported culture, fragile implementation motivation, time constraints, and fragmented record services) and facilitative actions (clarify capability requirements, create communication opportunities, and make discussion effective). The thematic synthesis process is provided in Supplement Appendix 3.

### Unsupported conditions

***Unsupported culture.*** What made healthcare professionals feel helpless was that in the current “malleable” society, the public sees death as not a natural part of life, and as a result patients’ final decision-making is affected by social forces and treatment needs and is bound by the concept of “if you don’t choose treatment, you will die.” [22, 25, 27] On the other hand, the family is a very strong unit, which plays an important role in decision-making, but family members tend to struggle with medical decision-making, leaving no opportunity to initiate ACP topics [23, 25, 26, 28, 29].

***Fragile implementation motivation***. Most healthcare professionals recognized the concept of ACP and were eager to provide guidance and support to patients, which led to a more collaborative approach to end-of-life management [17, 22, 24, 25, 27, 29, 31]. However, they had a negative attitude in practice, resulting in the lack of implementation motivation. Some healthcare professionals believed that ACP prevented them from rescuing patients, which is contrary to medical expectations to prolong life, and that initiating such a dialogue was tantamount to admitting medical failure and their own professional inadequacies [22, 25, 29]. Some argued that mentioning ACP made patients feel sad and that doctors were giving up on them, damaging the doctor-patient relationship, aggravating the negative emotions of patients, and causing a negative public perception of the government [22, 23, 27, 29–31]. They also found that they became emotional talking about the topic, overwhelmed by being too involved in patients’ deathbeds, and initially stressed in dealing with patients’ aggressive responses [27, 29, 31]. Some healthcare professionals felt that they did not have enough knowledge and experience, were afraid of being questioned by patients and family members, and worried that negative feedback would affect their professional confidence [24, 26, 29, 31].

***Time constraints.*** Healthcare professionals admitted that time constraints were an obstacle to initiating ACP. Some healthcare professionals believed that other issues, such as how to avoid various risks, occupied most of their time, and there was limited time to talk about ACP [31]. They only had time to talk about the general situation and not specific patient concerns, which did not solve all the problems plaguing patients [22, 25, 27].

***Fragmented record services.*** Healthcare professionals felt that the lack of a coordinated system hindered the implementation of ACP and led to fragmented record services [28]. Their ACP records were sloppy and failed to ensure the validity and suitability of the documentation [24]. In addition, the records were not specific enough, which led to conflicting understandings between family members and healthcare professionals on whether the relevant medical measures violated the wishes of the patients [23].

### Facilitative actions

***Clarify capability requirements.*** Healthcare professionals believed that it was important to have a clear understanding of what abilities they needed to have when implementing ACP. They needed to have strong communication skills, which depended on long-term experience accumulation and communication talent, and they also needed to master in-depth interviewing skills [23, 29]. Relevant skills such as reflection and ethical reasoning needed to be developed at the same time, rather than simply enforcing patients’ wishes [23]. Some knowledge of disease-related specificities was required to help guide patients on what may happen in the future; [17, 25, 26, 30, 31] for instance, explaining various symptoms and problems that could occur to patients in the future, taking preventive measures in advance for changes in patients’ future health status, providing information and support and making plans [17, 27, 31], enhancing insight into patients’ daily living ability, and satisfying their desires for daily living activities [23].

***Create communication opportunities.*** Healthcare professionals agreed that ACP communication opportunities could be flexibly created according to the actual situation. Some considered that it could occur as informal small conversations based on various responses to questions, similar to conversations about informed consent decisions and patients’ goals and treatment options [23, 25, 30, 31]. There were also some healthcare professionals who considered that when patients had serious diseases, poor prognoses, or negative behaviors, such as cancer patients who quickly associated their diagnosis with death, it was a good opportunity to talk about ACP [27, 30]. Utilizing home visits and long-term follow-up could also create multiple opportunities for discussion [22, 25]. Most agreed that various auxiliary communication tools, such as question prompt lists and high-quality leaflets with ACP information, could be used to help patients enhance their end-of-life thinking and ask questions to facilitate conversation initiation [22, 31].

***Make discussion effective.*** Healthcare professionals believed that ACP needed multidisciplinary team cooperation and that each member of the medical team should play a specific role in it to prevent information errors and lack of accountability, resulting in more positive responses [24, 25, 27, 28, 30]. It was proposed that interdisciplinary members such as dietitians, psychological counselors, and specialists should jointly discuss with patients about future care preferences [25]. Before initiating the topic, healthcare professionals should assess patients’ acceptance of ACP, use different approaches to deliver information, gauge patients’ understanding of the information from their retelling, and use personalized communication based on their understanding and education level [25]. In a frank way, they could also discuss clearly and openly when treatment may no longer be beneficial and the trade-off between quality and quantity of life [17, 29]. Healthcare professionals also recognized that patients’ preferences for future care depended on their own goals and values, avoided bringing in other perspectives, and encouraged patients to express their own ideas [27, 29] Respecting the wishes of patients should be updated at any time due to the impact of symptoms. [17, 23, 26, 27, 29]. Some healthcare professionals agreed that a communication template or predefined care guidelines that covered each patient’s palliative medical needs would be valuable to guide them in communicating effectively [17, 22, 31].

## Discussion

This paper utilized a meta-synthesis approach to review 11 studies involving the experiences and perspectives of healthcare professionals in providing ACP to life-limited patients. Two themes were identified: unsupported conditions and facilitative actions. The results showed that healthcare professionals perceived unsupported culture, fragile implementation motivation, time constraints, and fragmented record services as barriers to ACP implementation. In addition, they also proposed clarifying capability requirements, creating communication opportunities, and making communication effective as measures that could promote the development of ACP.

Healthcare professionals reported that talking about death induced feelings of anxiety and restlessness in patients [22, 27]. They felt that patients did not understand the relationship between ACP and treatment and assumed that mentioning ACP meant “dying soon” [29, 32]. Relevant social departments should make continuous efforts to improve public awareness of ACP and increase the sense of existence of ACP concepts, such as public education through advertising media and posters, and attempt to introduce ACP in municipal elderly care institutions and social service units to gradually increase social acceptance of ACP [17, 27, 33].

Whether in Asian countries or western countries, families played an important role in the whole decision-making process, which was similar to the results in other studies [5, 34, 35]. From the perspective of healthcare professionals, it was found that in addition to family members’ desire to lead decision-making, patients also relied on family members to make decisions [26]. This may have been due to the influence of familism and patients’ fear that making wrong decisions would go against their family’s wishes [26]. We found that in Japan, healthcare professionals viewed family members as “key referents” and had end-of-life care discussions with them before giving patients an informed choice [23]. This finding is in line with Martina et al., who suggested that healthcare professionals in Asian countries tended to give a greater voice to family members [5]. In contrast, in the United Kingdom and Finland, most healthcare professionals were more likely to comply with patients’ wishes when decisions conflicted with those of family members [17, 24]. These differences may have been influenced by the collectivist culture in Asia, in which healthcare professionals tend to maintain harmony with family members [36, 37]. Since patients become less active as the disease progresses, family members need to play a more central role in communication [38]. The involvement of family members can contribute to goal-harmonious care and reduce the burden of decision-making to better leverage the value of ACP [38]. Therefore, healthcare professionals need to find a balance of interests and explore a way to maintain harmony between themselves, patients, and their families. Thus, a healthcare professional-initiated, patient-centered, and family-oriented discussion approach may be worth promoting.

Healthcare professionals were eager to achieve patient-centered care goals but were concerned about the negative impact of ACP, which was similar to the findings of Keijzer et al [22, 27, 29, 31, 39]. Healthcare professionals also reported their unease about discontinuing patients’ life-sustaining treatment and uncertainty about what the law provided them for protection [27, 31]. Moreover, the problem of unimplemented funds further weakened their motivation [29, 31]. Therefore, improving the legal system and providing financial support can increase the motivation of healthcare professionals [31, 40, 41].

Clear records are the premise of respecting the wishes of patients [29]. Documents with a structured and legal effect protect patients’ wishes from being violated [17, 24, 42]. Establishing a coordinated and shared ACP system can improve the continuity of patient-related information management and the effectiveness of documentation, as well as ensure that patients’ ACP documentation can be legally transferred to other care facilities [23, 28, 31]. It was suggested that document templates should be embedded into the system to provide clear ACP instructions, reduce the variability of documents, and collect high-quality ACP information [43]. The contents of the records should generally include who participated in the discussion, who was the surrogate decision-maker for patients, patients’ goals and values, prognosis, treatment intention, and expected outcome of treatment [44, 45]. Considering the differences in the culture and legal system of each country, we suggest that the system should be tailored to meet different needs. In addition, the system should be used to help identify untreatable patients, alerting healthcare professionals to timely initiate ACP with them.

Healthcare professionals believed that they needed multiple competencies to successfully implement ACP [17, 23, 25, 26, 29, 31]. However, due to the lack of systematic education and training courses, they had not acquired sufficient knowledge and skills, so they did not have the confidence to do it [29, 31]. Strengthening ACP training is an essential measure to improve the confidence and willingness of healthcare professionals [46]. Educational resources should be considered in the form of learning communication skills, role playing, webinars, and observational learning [28, 47]. The trainees should also agree to ACP themselves to deeply experience the feeling of the ACP process [22].

Healthcare professionals in different countries judged the timing of ACP initiation differently [5]. There is a large degree of uncertainty about the trajectory of the disease, which healthcare professionals dislike and have difficulty acknowledging to their patients, thus affecting the timing of ACP initiation [42]. Compared with cancer, the life-limiting nature of chronic diseases was not obvious, patients were less likely to think about death, and it was difficult for healthcare professionals to judge the appropriate time to initiate the topic [22, 27, 30, 40]. Most studies recommended that patients with chronic diseases should be introduced to ACP as soon as possible [25, 30, 40]. This study integrated the views of healthcare professionals on the timing of ACP initiation for patients with multiple life-limiting disease types and found that ACP could be initiated in any informal form and the timing could be flexible [23, 25, 30, 31]. Moreover, the initiation of ACP should be based on the nature of the patient’s disease and response to the disease situation [27, 28, 30]. To increase the frequency of this conversation, an auxiliary tool that can enlighten patients to think about end-of-life issues and encourage them to ask questions is particularly important [22, 31].

Martina et al. showed that physicians and oncologists were more involved in ACP than nurses [5]. However, after integrating the views of different healthcare professionals, we found that ACP should be a shared interdisciplinary responsibility, and healthcare professionals of different professions should play different roles according to their own work nature and strengths. For example, oncologists and physicians should be responsible for treatment decisions and sharing important clinical information to help patients define treatment goals [17, 28], while nurses could use their communication skills to discuss decisions with patients and their families [17, 25], and the psychology team could address patients’ emotional and mental health issues [28]. Moreover, primary care providers should be more proactive in bringing up the topic because of their long-standing relationship with their patients, and palliative care specialists should act as mediators between hospitals and primary care [23, 28]. This interaction between healthcare professionals could solve the lack of time for discussing ACP and avoid the patient receiving multiple conflicting information amid competing clinical responsibilities [28]. Eliciting patients’ values and preferences is an important step in successful ACP communication [48, 49]. Interdisciplinary teamwork can provide effective information and assess needs for patients more comprehensively and individually, as well as facilitate them to express their ideas [24, 27, 28, 30, 50, 51]. Moreover, Cottrell et al. intriguingly found that if healthcare professionals have a trusting and empathic relationship with patients, patients will feel empowered and more willing to engage in ACP, which is also important for facilitating their expression of ideas [52]. Overall, ACP is a communication process that must be fluid to allow for updating patients’ wishes [17, 23, 26, 27, 29, 49].

### Strengths and limitations

This study is the first systematic review to integrate the experiences and perspectives of different healthcare professionals in different care settings on the provision of ACP for life-limited patients. The healthcare professionals in this review came from a variety of healthcare professions, including general practitioners, doctors, nurses, and medical specialists in different fields, which allowed us to gain a unique perspective on the complexities of implementing ACP. The review included their views on providing ACP for life-limited patients with advanced cancer, dementia, chronic kidney disease, heart failure, end-stage renal disease, and severe chronic obstructive pulmonary disease, which provided rich information. The 11 studies included nine countries in total, covering North America, Europe, Oceania, and Asia, each with diverse cultures. In addition, this review used the JBI Critical Appraisal Checklist for Qualitative Research to strictly evaluate the quality of the included studies and conducted a rigorous, thematic, integrated analysis of the findings.

This review has several limitations. First, limiting the search to studies published in English and Chinese may have excluded important studies in other languages, potentially depriving our review of valuable contributions. Second, there were relatively few studies included in this review, and most did not explain the researchers’ own situation from the perspective of culture, values, or theory, which may have affected the comprehensiveness of the integration and the interpretation of the integrated results. Third, only one of the included studies was rated A, and the rest were all rated B, indicating that the quality of the included studies was not high. Finally, we did not limit the care settings and extensively discuss the experiences and views of healthcare professionals on providing ACP to patients with LLI. There may be healthcare professionals with different medical occupational types or different working environments who have different views on the implementation of ACP, but this was also the advantage of this review, which integrated the views of different healthcare professionals and produced richer and diversified information.

## Conclusion

This article systematically reviewed the experiences and perspectives of healthcare professionals on providing ACP to life-limited patients and explored the barriers to and need for implementing ACP. Relevant departments should create a cultural environment suitable for ACP and improve healthcare professionals’ motivation by strengthening both the legal system and financial support. Healthcare systems need to establish a coordinated and shared system to improve the continuity of patient-related information management and the effectiveness of documentation, increase the knowledge and skills of healthcare professionals through educational training, and promote multidisciplinary collaboration. Future research should compare the differences in the needs of healthcare professionals in different cultures when implementing ACP to develop systematic implementation guidelines tailored to those cultures.

## Electronic supplementary material

Below is the link to the electronic supplementary material.

Appendix 1: A search strategy in Embase

Appendix 2: Consistency of quality assessment of qualitative studies

Appendix 3: The process of thematic synthesis

## Data Availability

Not applicable. The research strategy, the list of the included articles and the process of data synthesis are in the Table and Supplement Appendix. The data could be made available upon reasonable request from the corresponding authors.

## Abbreviations

ACP: Advance care planning
AHPs: Allied health professionals
ALS: Amyotrophic lateral sclerosis
CKD: Chronic kidney disease
CNKI: China National Knowledge Internet
COPD: Chronic obstructive pulmonary disease
CPN: Community psychiatric nurse
GPs: General practitioners
HF: Heart failure
LLI: Life-limiting illness
