# Supplementary material for: Experiences and perspectives of healthcare professionals implementing advance care planning for people suffering from life-limiting illness: a systematic review and meta-synthesis of qualitative studies
Source: BMC Palliat Care. 2023 May 6;22:55. doi: 10.1186/s12904-023-01176-7 (PMC10163819; doi:10.1186/s12904-023-01176-7)
Supplement: Supplementary file 1 — Appendix 1: A search strategy in Embase [file 12904_2023_1176_MOESM1_ESM.docx]

**Appendix 1.** A search strategy in Embase.

| Search Processes | Results |
| --- | --- |
| Step 1: 'advance care planning'/exp OR 'advance care plan*':ti,ab OR 'advance healthcare plan*':ti,ab OR 'advance health care plan*':ti,ab OR 'advance medical plan*':ti,ab OR 'advance medical direct*':ti,ab OR 'advance care directive*':ti,ab OR 'advance directive*':ti,ab OR 'advanced care plan*':ti,ab OR 'advanced healthcare plan*':ti,ab OR 'advanced health care plan*':ti,ab OR 'advanced medical plan*':ti,ab OR 'advanced medical direct*':ti,ab OR 'advanced care directive*':ti,ab OR 'advanced directive*':ti,ab | 12,393 |
| Step 2: 'medical staff'/exp OR 'medical staff*':ti,ab OR 'medical personnel*':ti,ab OR 'medical professional*':ti,ab OR 'medical worker*':ti,ab OR 'health staff*':ti,ab OR 'health personnel*':ti,ab OR 'health professional*':ti,ab OR 'health worker*':ti,ab OR 'health provider*':ti,ab OR 'medical care provider*':ti,ab OR 'medical care personnel*':ti,ab OR 'medical staff member*':ti,ab OR 'healthcare professional*':ti,ab OR 'healthcare staff*':ti,ab | 228,505 |
| Step 3: Step 1 AND Step 2 | 933 |
