# Supplementary material for: Experiences and perspectives of healthcare professionals implementing advance care planning for people suffering from life-limiting illness: a systematic review and meta-synthesis of qualitative studies
Source: BMC Palliat Care. 2023 May 6;22:55. doi: 10.1186/s12904-023-01176-7 (PMC10163819; doi:10.1186/s12904-023-01176-7)
Supplement: Supplementary file 2 — Appendix 2: Consistency of quality assessment of qualitative studies [file 12904_2023_1176_MOESM2_ESM.docx]

**Appendix 2.** Consistency of quality assessment of qualitative studies.

|  | C1 | | C2 | | C3 | | C4 | | C5 | | C6 | | C7 | | C8 | | C9 | | C10 | | Agreement |
| --- | --- | --- | --- | --- | --- | --- | --- | --- | --- | --- | --- | --- | --- | --- | --- | --- | --- | --- | --- | --- | --- |
|  | NX -Z | LY | NX -Z | LY | NX -Z | LY | NX -Z | LY | NX -Z | LY | NX -Z | LY | NX -Z | LY | NX -Z | LY | NX -Z | LY | NX -Z | LY |  |
| Wichmann et al.^27^ | Y | Y | Y | Y | Y | Y | Y | Y | Y | Y | U | Y | U | U | Y | Y | Y | Y | Y | Y | 0.9 |
| Toguri et al.^28^ | Y | Y | Y | Y | Y | Y | Y | Y | Y | Y | U | U | U | Y | Y | Y | Y | Y | Y | Y | 0.9 |
| Sellars et al.^29^ | Y | Y | Y | Y | Y | Y | Y | Y | Y | Y | U | U | U | U | Y | Y | Y | Y | Y | Y | 1 |
| Schichtel et al.^22^ | Y | Y | Y | Y | Y | Y | Y | Y | Y | Y | Y | Y | Y | Y | Y | Y | Y | Y | Y | Y | 1 |
| Robinson et al.^24^ | Y | Y | Y | Y | Y | Y | Y | Y | Y | Y | U | U | Y | U | Y | U | Y | Y | Y | Y | 0.8 |
| O’hare et al.^25^ | Y | Y | Y | Y | Y | Y | Y | Y | Y | Y | U | U | Y | Y | Y | U | Y | Y | Y | Y | 0.9 |
| De Vleminck et al.^30^ | Y | Y | Y | Y | Y | Y | Y | Y | Y | Y | U | U | U | Y | Y | U | Y | Y | Y | Y | 0.8 |
| Menon et al.^26^ | Y | Y | Y | Y | Y | Y | Y | Y | Y | Y | U | U | Y | Y | Y | U | Y | Y | Y | Y | 0.9 |
| Manthorpe et al.^31^ | Y | Y | Y | Y | Y | Y | Y | Y | Y | Y | U | U | U | U | Y | U | Y | Y | Y | Y | 0.9 |
| Kuusisto et al.^17^ | Y | Y | Y | Y | Y | Y | Y | Y | Y | Y | U | U | Y | Y | Y | Y | Y | Y | Y | Y | 1 |
| Hirakawa et al.^23^ | Y | Y | Y | Y | Y | Y | Y | Y | Y | Y | Y | Y | U | U | Y | U | Y | Y | Y | U | 0.8 |
|  |  |  |  |  |  |  |  |  |  |  |  |  |  |  |  |  |  |  |  |  | Mean=0.9 |

C1=Congruity between the stated philosophical perspective and the research methodology.

C2=Congruity between the research methodology and the research question or objectives.

C3=Congruity between the research methodology and the methods used to collect data.

C4=Congruity between the research methodology and the representation and analysis of data.

C5=There is congruence between the research methodology and the interpretation of results.

C6=Locating the researcher culturally or theoretically.

C7=Influence of the researcher on the research.

C8=Representation of participants and their voices.

C9=Ethical approval by an appropriate body.

C10=Relationship of conclusions to analysis or interpretation of the data.

U=unclear; Y=yes.
