# Supplementary material for: Experiences and perspectives of healthcare professionals implementing advance care planning for people suffering from life-limiting illness: a systematic review and meta-synthesis of qualitative studies
Source: BMC Palliat Care. 2023 May 6;22:55. doi: 10.1186/s12904-023-01176-7 (PMC10163819; doi:10.1186/s12904-023-01176-7)
Supplement: Supplementary file 3 — Appendix 3: The process of thematic synthesis [file 12904_2023_1176_MOESM3_ESM.docx]

**Appendix 3.** The process of thematic synthesis.

| Analytical Themes  (Themes and Subthemes) | Descriptive Themes | Initial Codes | | Illustrative Quotes |
| --- | --- | --- | --- | --- |
| **Theme1: Unsupported conditions** | | | | |
| Subtheme 1: Unsupported culture | Social culture regards death as taboo | | Death is not a natural part of life | In my opinion, death nowadays is not experienced as being part of your life anymore…. People find it hard to talk about it. Which makes it difficult for me as [a] GP.^26^ |
|  |  |  |  | That’s the biomedical approach which I think still sees death as a failure, as an enemy rather than a natural part of life.^21^ |
|  |  |  | Patients fear death under the influence of social culture | I just had one patient who was, like, 85 and he ended up on dialysis ... I don’t think the decision was, I don’t know, it just didn’t go like you would hope it would for someone that age. I don’t think he was presented with all of his options, in terms of not doing anything. I think he was only presented with, like, “You have to do this, otherwise you’ll die.” ^24^ |
|  | Family members’ decision hinders ACP implementation | | Family members dominate decision-making | And if you want to discuss further, for example options of chemo, or other treatment, then he will say: “Oh, please talk to my son.” You know, it’s like [with] everything, go speak to them.^25^ |
|  |  |  |  | So that is more patients in the ICU, and we are not there to make those decisions, because when the patients are in ICU, families make those decisions.^24^ |
|  |  |  | Family members insist on life maintenance treatment | You know, one of the difficulties is the patient who comes in here and their family wants to be aggressive.^27^ |
|  |  |  |  | We were very strong in suggesting stopping dialysis to one patient because she was pretty much out of it and [her family] took great objection to that.^28^ |
|  |  |  |  | Some families ask us to continue to feed their loved ones even when they experience dysphasia and are at risk of choking or developing pneumonia.^22^ |
| Subtheme 2: Fragile implementation motivation | Recognition of the idea | | Help patients make a perfect curtain call for their lives | It brings peace and tranquility. Also, I think, people will reflect on their situation more...they do not leave everything to the last minute, they arrange things in advance, talk about it, are able to say farewell.^26^ |
|  |  |  | An opportunity to protect the patient’s wishes from being violated by others | With this kind of a chronic disease process and things that might happen, it's a good thing to...look at what options might be available for you later, if things progress or become worse, you know, make your wishes known, so that somebody won’t disrespect them.^24^ |
|  |  |  | Help with hospice management | Nurses have for a very long time felt powerless around end-of-life management and they see advance care planning as a way of participating and helping the patients, not just through life but hopefully help them have a comfortable death.^28^ |
|  |  |  | Offers a wider range of health and is synonymous with holistic care | I think for GPs on the whole, the thing that floats our boat, is the idea of holistic care. It is the idea of not just firefighting the moment, but actually thinking about a broader definition of health.^21^ |
|  |  |  | Give the patient and family members support and security to cope with the disease | And it's also like a really important part of the patient and family members always knowing where to turn to if something comes up, that's like the crucial thing. It also brings a sense of security.^16^ |
|  |  |  | Provide guidance and support for dementia patients who will lose decision-making ability in the future | [...] it couldn't be more relevant to a patient group [...] we know that their capacity gets affected later on in life as their dementia progresses, so being able to make plans early on and helping them do that can really help them later on.^30^ |
|  |  |  | Ideal opportunity for patients to open up and ease their fears | They raise any fears that they may have. So it's an ideal opportunity.^23^ |
|  | Negativity towards practice | | Prevent resuscitation of patients | The nurses were trying to stop me. They had had this lecture on advance care planning and how it was illegal to resuscitate people. I remember being really quite upset that this nurse was trying to stop me resuscitating a young patient.^28^ |
|  |  |  | Against medical expectations | That clashes with the expectation of medicine—that medicine is about curing problems, about extending life. Nobody is stepping back, saying, “You are dying, and shall we plan for that so you have a good death?”^21^ |
|  |  |  | Admit the failure of medicine | So the physicians I think they sometimes look on this as a failure. They kind of keep pushing forth.^24^ |
|  |  |  |  | I think it's very difficult if your lifetime approach has been around successes and length of life. It’s almost an acknowledgement of the failure of medicine, isn’t it, to have that conversation.^21^ |
|  |  |  | Damage the doctor-patient relationship | My biggest fear was getting it wrong and upsetting the person and not being able to recover that and damaging the relationship and not being able to go there again.^21^ |
|  |  |  |  | I once had a patient who stopped visiting me because I had tried to talk about the end of life....^26^ |
|  |  |  |  | Patients will often perceive when you raise these issues that you are actually giving up on them and planning for their death and that you’re giving up on active treatment.^28^ |
|  |  |  | Aggravate patients’ negative emotions | I end up sometimes having to say, “Okay, let’s not go there. We’ll do this another time,” or upsetting people and making them go out thinking they are about to die.^28^ |
|  |  |  |  | Patients experiencing severe dyspnea may feel like they’re about to die, so when ACP discussions are initiated with them at that time, they might think that they are being handed a death sentence.^22^ |
|  |  |  |  | ...people are worried enough getting a dementia diagnosis, but then to think, you know, “Well you’re saying, well I shouldn’t go to [the] hospital?"...because people get sick and what do we think? We think we need the hospital...I think that it’s just worrying the person....^30^ |
|  |  |  |  | “It’s time that you do something about it, that you start planning this,” I think it’s a bit of a taboo to start discussing this, to tell someone with Alzheimer’s, in a year you won’t know what you’re doing anymore.^29^ |
|  |  |  | Cause negative public perception of the government | What is the government’s objective? Are they concerned I’m getting too old? Am I too expensive?^26^ |
|  |  |  | Bring emotional burden to healthcare professionals | Sometimes you have deathbeds of which you think, “I'm rather getting swept up in it too much....” People confide in you so much, you’re so intimate, that I sometimes feel it is a little too much for me.^26^ |
|  |  |  |  | If you have somebody who is aggressive in their responses when it’s introduced, it’s quite stressful because there is room for conflict, and I find I still feel a bit stressed about having to introduce the topic to people depending on what that initial reaction is.^28^ |
|  |  |  |  | It’s kind of thinking to the future, thinking to when they’ll be in a less good place than they are now, and some people shy away from doing that.^30^ |
|  |  |  | Lack of knowledge and fear of being questioned | I don’t think I really know enough to actually do it...(the) relative might even have more advanced knowledge and questions they want to ask; I might not be able to give the answer.^30^ |
|  |  |  |  | Because that one I think - planning what sort of treatment that I am going to have ... till if I get (into) accident. Then if I have an advance medical directive so they decide not to resuscitate me. If I have an advance care plan, whereby I plan what are the things that I want towards the end-of-life, then will there be confusion between these two?^25^ |
|  |  |  |  | I get confused about the terminology about advance care and advance directive and that and one's legally binding and one isn't and it all becomes a bit of a blur.^23^ |
|  |  |  | Negative feedback affects professional confidence | Even now after nearly five years simply because you just don't know what reaction you are going to get and when you really believe in something you don’t want to get negative feedback.^28^ |
| Subtheme 3: Time constraints | Other clinical events take up most of the time | | Other competitive issues and avoidance events about various risks have attracted more attention | I just wish we had more time...it’s something that isn’t addressed as it should be really, because of all the other competing issues.^30^ |
|  |  |  |  | There is often a focus on risk and reducing risk and avoiding risk...sometimes it's not the most pressing of things to be talking about.^30^ |
|  | Not enough time for ACP to work | | Unable to listen to patients | If you are too busy yourself, how could you be able to listen? In that case you could ask your questions, but how could you be able to really listen to the patient? Or do something through unspoken communication?^26^ |
|  |  |  | In a short time, can only talk about the big picture | You are not going to do ACP in ten minutes. It’s going to take you two hours to do this properly. You just have to make it work. I know it’s difficult.^21^ |
|  |  |  |  | I tend to think that my discussions with the patients...are important for them.... I think I can give them “the big picture”; however, my time is limited, so.... I always am able to give my opinion and allow them to ask some questions, but in the short period of time, it doesn’t address everything.^24^ |
| Subtheme 4: Fragmentated record services | Unable to ensure the validity and suitability of documentation | | The records are misleading | When long-term feeding is medically required, withholding or withdrawing tube feeding may be considered as undertreatment and an ethical issue. However, in some cases, family members view tube feeding as life support, and as against their loved one’s wishes.^22^ |
|  |  |  | Sloppy records | It was a document that had been written about three years ago of someone who had motor neurone who was having a fit...staff were saying, “We don’t want any investigations, no, look, we've got this,” but actually when you looked at it, it was so non-specific or it wasn’t legally binding in any stretch of the imagination…it wasn’t worth the paper it was written on.^23^ |
|  | Lack of coordinated recording systems | | Record dispersion | There should be one source of documentation, right.... That we have that advance care planning documentation on there so that with one click, we know. Right now we have to go to five or six different health records, and then we still may not get it.^27^ |
| **Theme 2: Facilitative actions** | | | | |
| Subtheme 1: Clarify capability requirements | Communication skills | | Long-term experience accumulation and communication talent | That has to be an experiential thing. I think I'm successful but it’s something that I know I’ve gradually improved upon over time. I think it’s heavily personality driven and it’s also heavily dependent upon inherently having good communication skills.^28^ |
|  |  |  | In-depth interview techniques | I think that healthcare professionals engaging in ACP communication must develop in-depth interview skills.^22^ |
|  | Ethical reasoning ability | | Learn to reflect | From an ethical standpoint, good care managers consider both the end-of-life care options that the clients and families choose, and the motivations behind these decisions.^22^ |
|  | Learn about medical expertise | | Provide relevant knowledge of the disease and help patients analyze the prognosis under different options | If the Advance Decision (AD) is about some medical treatment or withdrawal of treatment, a bit more understanding and knowledge about that specific condition [...] the prognosis of that condition with and without treatment.^30^ |
|  |  |  |  | For a good planning you need to be well informed, you need to know what are the possibilities and what aren't the possibilities.^29^ |
|  |  |  |  | I guess it's more like, when you know in a way, on a general level, what certain cancers and illnesses involve and know something about what the average care pathway looks like as it were, well then you can perhaps give the patient some guidance on what may lie ahead.^16^ |
|  |  |  |  | It's an opportune time, no matter where you work, to be able to talk to patients about it, but again, you have to .... have some practice and knowledge about how to approach it, so it's not going to be threatening to them.^24^ |
|  |  |  |  | So the whole ACP idea is that information, stage, cause of the disease and treatment options are adequately provided to the family members and the patient. This will be helpful, in terms of contributing to the whole ACP, make it more advantageous, beneficial and more useful to the subsequent users.^25^ |
|  | Proactive ability | | Think ahead about the patient’s future symptoms and problems | If there are urinary tract infections? But in a way that’s also being proactive, they are small simple things, but for nurses they mean being proactive.^16^ |
|  |  |  | Know the barriers to conversation in advance | If you feel that there is resistance [in talking about the end of life], it is better you know it up front instead of in the last week of life.^26^ |
|  |  |  | Provide information and planning support | Usually I would just print something off for them to read, Alzheimer's Society...say that “You can go to the Citizen’s Advice or Age UK, you know for help with the form”...nowadays we assess, signpost, and discharge.^30^ |
|  | Observation ability | | Enhance insight into the patient’s ability to live daily | Home-visit nurses focus on supporting their clients’ wishes regarding activities of daily living, like eating, bathing, and toileting, rather than on life-sustaining treatments.^22^ |
| Subtheme 2: Create communication opportunities | Conversations that can occur informally anytime, anywhere | | Small conversation in response to questions | It [ACP] does not have to be anything formal, it may also be just a little chat in response to...I think it is very important that that is monitored.^29^ |
|  |  |  | Conversations discussed between the lines | We will very often discuss as things go down the line, you know, “Where do you want to be, who do you want to look after you?”, that, that sort of thing.^30^ |
|  |  |  | Similar to discussing informed consent decisions | Physician-led ACP, which is commonly practiced, bears [a] resemblance [to] informed consent decisions.^22^ |
|  |  |  | Ongoing conversation about patients’ goals and treatments | We actually, in dialysis, probably do a fair amount of it...we...constantly talk about whether people are going to want dialysis and their choices, including no treatment...especially with people...who have a lot of comorbidities, and we will talk about not having to do dialysis to extend life. So, I think it’s emblematic of what advance care planning is...an ongoing conversation about patients’ goals and treatments.^24^ |
|  | Identify communication opportunities based on the patient’s behavior | | The emergence of negative patient behavior is the time to discuss ACP | When they present their will, having downloaded a lot of information, thinking, this is it, I have done what I had to do...that's not how it works, of course. But it is a nice occasion to start discussing these matters.^26^ |
|  |  |  | Cancer patients tend to associate with death after diagnosis | For cancer it’s known, “cancer—death,” people do make that association.^29^ |
|  | Create time for discussion | | Long-term patients discuss multiple times through follow-up | If it’s a patient whose been a long-term individual...whom I’ve followed for...several years, it’s actually quite easy, because we’ve been talking about this here and there over the course of multiple different appointments.^24^ |
|  |  |  | Use home visits to create communication time | I think having two hours after surgery to go and see this family was vital to have a successful end-of-life conversation. I see these kinds of patients after work or on the way home. It was helpful to have as much time as we needed to discuss these things. We continued with the discussion until it naturally came to an end. This is incredibly valuable.^21^ |
|  | Provide auxiliary communication tools for patients to facilitate conversation initiation | | Provide ACP related information to help patients think | We could just give information out to people to be able to reflect a little bit more on at the end of the assessment [...] to have something about advanced care planning [...] it would make it a lot easier for people.^30^ |
|  |  |  | Provide question prompt lists to facilitate doctor-patient discussions | I think that shared decision-making tools would be excellent. Not least of all to facilitate the conversation, because it is so much easier. So, that’s one of the pluses of a form. You could give it to the patient in advance and they could write quite a bit on it. And that gives them the opportunity to put as much or as little, and tick boxes about what they did or didn’t want to discuss. That really helps the clinician to know where the patient is at.^21^ |
| Subtheme 3: Make discussion effective | Multidisciplinary cooperation brings positive responses | | Early teamwork improves patient satisfaction and quality of life | It’s always proven that the early involvement of a supportive care team or a palliative care team has such a huge impact in terms of patient satisfaction but also in terms of management issues in terms of quality of life and even in terms of survival.^27^ |
|  |  |  | Multiple different professionals play different roles to complete | Or maybe, I don’t know, a nurse, a dementia adviser.... It’s probably not only one role: it’s psychiatrist, it’s nurse, you know it’s several where you can’t just give it to one professional.^23^ |
|  |  |  | Involvement with medical specialists to make an impact | I feel that you almost need to have a personal relationship with a specialist before you can have any impact.^29^ |
|  |  |  | Medical specialists can discuss with general practitioners when making plans for patients | They [medical specialists] are better informed about further possibilities and I see what happens at home.... Very often a patient is discussed in the multidisciplinary consultation in the hospital. Maybe they could also consult the GP more often: these are our plans, do they seem possible to you, too?^26^ |
|  |  |  | Multidisciplinary cooperation to discuss preferences with patients | Then we have a program when people start reaching stage 4 or 5, when they’re [going to] need dialysis, that they come to education classes. And we have our staff, our dietician, social worker, transplant coordinator, all those people there, who talk to them about options, but also start planting the seeds about thinking about advance directives and what kind of care they might want to see.^24^ |
|  | Ensure the effectiveness of information transmission | | Deliver the information in different ways and ask the patients to repeat it | I think that, very commonly, the time when the clinician is ready to share the information has nothing to do with when the patient is ready to hear the information. That is the schism. You know I think it leads to all kinds of “well, I told them about this”.... So, tell them again. Tell them a different way. Ask them to tell you what they just heard. Find out what they understood of what you just said. Because frequently, when people are freaked out...they hear the first word of every sentence.^24^ |
|  |  |  | Adopt a personalized approach to communication | I think it’s really that literacy issue and understanding what kind of intellect this person is in possession of, so that you are speaking to them in a way that makes sense to them.^24^ |
|  | Tell the truth about the illness | | Clearly and openly discuss and weigh options | How do I help the patient with this discomfort? Well, one of the things that I do is I try to be clear: “Sometimes people facing this problem, with kidney disease or on dialysis, sometimes people die. Well, how do you feel about that?”^28^ |
|  |  |  |  | So that our professional expertise is utilized as it were, so that we assess, together with the patient, if there is a need to move on to some sort of treatment.^16^ |
|  |  |  | Be honest | if we're honest and open with them, they're honest and open back to us.^28^ |
|  | Ensure that patients' wishes depend on their own goals and values | | Avoid bringing in ideas | Not because you want to outline all possible scenarios, but because you know what kind of scenarios patients might expect.^26^ |
|  |  |  |  | If you're asking closed questions you're bringing your values and opinions into it. I suppose there's quite a few patients that will ask, “What do you think I should do?” but it's not about what you think they should do, it's what they want to do. It's all, you know, that can be done and what they want.^28^ |
|  |  |  | Encourage patients to express themselves | I used to be more reactive; now I try to take a more proactive stand…Before, I would say, “No, you’re not doing too well, but we will make the best of it.” Now, I would more likely say, “Well, try to think about it yourself, how do you envisage it?”^26^ |
|  | Ensure that patients’ wishes can be updated | | Patients’ wishes will change with the impact of symptoms | It is the same as having children: you have very specific ideas about that, about delivering at home, and breastfeeding and all that. You would not be the first to say, “'ll deliver comfortably at home and in the bath,” and that by the time you have contractions the nurse says, “Well, let’s step into a nice bath,” and you say, “A bath?! I don’t want to step into my bath. No way. I want to go to the hospital, now!”^26^ |
|  |  |  |  | Certain clients with severe COPD who had originally wished to die at home, later chose to be hospitalized when they began experiencing unbearably severe dyspnea.^22^ |
|  |  |  |  | We often see patients, who come in with symptoms from their kidney failure and then they are breathless at that point of time, when they are actually actively suffering and dying from the illness, they start to change their minds.^25^ |
|  |  |  |  | The care plan is then updated at every encounter.^16^ |
|  |  |  | Respect the change of patients’ wishes | It’s important that you don't make patients feel locked into the decision. They need to feel comfortable with it so it applies all the way through that continuum: “Let me just reassure you that if you change your mind you need just to let us know.”^28^ |
|  | Use auxiliary tools to improve the effectiveness of communication | | Communication templates help complete the discussion | When you are discussing a patient, a template that covers each individual patient’s palliative care needs, which can be completed during the meeting that would be a useful tool.^21^ |
|  |  |  | Pre-defined care guidelines guide the direction of implementation | It’s a help, it helps that it has been done and been discussed, a really great help, so you know which way the treatment is going and what will and will not be done.^16^ |
|  |  |  | Framework can improve medical staff mobility | If I, if I was sort of given, given a framework to do that in, I would very happily do it.^30^ |
